# P2X7R and P2X4R expression of mice submandibular gland in high-fat diet/streptozotocin-induced type 2 diabetes

**DOI:** 10.1038/s41598-024-60519-3

**Published:** 2024-05-13

**Authors:** Jiratchaya Srisutha, Ippei Watari, Masato Akakura, Minami Watanabe, Chidsanu Changsiripun, Takashi Ono

**Affiliations:** 1 Department of Orthodontic Science, Graduate School of Medical and Dental Sciences, Tokyo Medical and Dental University (TMDU), Yushima 1-5-45, Bunkyo city, Tokyo, 113-8510 Japan; 2 Department of Orthodontics, Faculty of Dentistry, Chulalongkorn University, Bangkok, 10330 Thailand

**Keywords:** Submandibular gland (SMG), Type 2 diabetes mellitus (T2DM), P2X7 purinergic receptor (P2X7R), P2X4 purinergic receptor (P2X4R), Diabetes, Immunochemistry

## Abstract

Type 2 diabetes mellitus (T2DM) is a chronic inflammatory disease that can compromise the functioning of various organs, including the salivary glands (SG). The purinergic system is one of the most important inflammatory pathways in T2DM condition, and P2X7R and P2X4R are the primary purinergic receptors in SG that regulate inflammatory homeostasis. This study aimed to evaluate P2X7R and P2X4R expression, and morphological changes in the submandibular gland (SMG) in T2DM. Twenty-four 5-week-old mice were randomly assigned to control (CON) and diabetes mellitus (DM) groups (n = 12 each). Body weight, diet, and blood glucose levels were monitored weekly. The histomorphology of the SMG and the expression of the P2X7R, and P2X7R was evaluated by immunohistochemistry (IHC) staining and reverse transcription-quantitative polymerase chain reaction (RT-qPCR) at 11 and 13 weeks of age. Our findings indicate a significant increase in food consumption, body weight, and blood glucose levels in the DM group. Although a significant increase in P2X7R and P2X4R expression was observed in the DM groups, the receptor location remained unchanged. We also observed a significant increase in the acinar area in the DM13w group, and a significant decrease in the ductal area in the DM11w and DM13w groups. Targeting purinergic receptors may offer novel therapeutic methods for diabetic complications.

## Introduction

Diabetes mellitus (DM) has been reported by the International Diabetes Federation as one of the most prevalent diseases since 2019. According to the World Health Organization (WHO), approximately 3.5% of deaths related to non-communicable diseases are attributed to type 2 DM (T2DM), which is predominantly caused by lifestyle factors, such as obesity^1,2^, and genetic factors^3^. T2DM is a chronic inflammatory disease that can compromise various organ functions throughout the body, leading to kidney disease, arteriosclerosis, neuropathy, candidiasis, and glandular dysfunctions, particularly in the salivary glands (SG)^3^. Previous studies have evaluated the influence that T2DM can have on the development of inflammation related to transforming growth factor β1 (TGFβ-1) expression^4^ as well as apoptosis in SG parenchymal cells^5^. Dyslipidemia is a characteristic feature of DM^6^. Previous studies in humans have revealed that prolonged consumption of a high-fat diet causes T2DM, leading to high lipid accumulation in the SG. High levels of saturated fatty acids in plasma can cause SG cells to exhibit increased interleukin (IL)-6 production and may lead to the development of inflammation^7^. Consequently, excessive SG inflammation and degeneration of SG cells affects the function of SG in terms of saliva production^8^ and composition^9^. When SG function is impaired, xerostomia or dry mouth can develop, which is a common complication in T2DM patients^8^.

Several factors cause SG dysfunction, such as Sjögren’s syndrome (SS), radiography of the head and neck area, aging, medications, and even DM, which leads to the destruction of saliva-producing cells that fail to restore themselves^10,11^. Current treatments for abnormal saliva secretion are focused on muscarinic receptor agonists (i.e., pilocarpine) or the use of artificial saliva to relieve symptoms^12^. However, one of the causes of SG dysfunction is inflammation, which is a characteristic of DM, a chronic inflammatory disease. Therefore, solving the root cause by focusing on the inflammatory process that causes SG damage may help sustain the management of this complication^13^.

One of the most important inflammatory signaling pathways is the purinergic system, which is responsible for controlling the inflammatory process in an increased ATP environment^14^. In the SG, the purinergic receptor subtypes that control the inflammatory process are the ionotropic P2X4 and P2X7 purinergic receptors (i.e., P2X4R and P2X7R)^15^. Although previous studies on purinergic signaling in different organs (e.g., pancreas, brain, kidney, eyes, skin, bladders, and gastrointestinal organs)^16^ have been performed extensively in various immune disorders (i.e., SS and diabetes), studies targeting purinergic receptors, specifically P2X7R and P2X4R, in the SG of patients with diabetes remain scarce. The distinct functionalities of P2X4R (capable of both pro- and anti-inflammatory responses) and P2X7R (primarily pro-inflammatory)^17^ necessitate investigation of both receptors to gain a comprehensive understanding of purinergic signaling in T2DM-related salivary gland dysfunction. In this regard, paying more attention to purinergic signaling in the SG is expected to help understand the comprehensive mechanisms and develop a novel therapeutic approach for targeting purinergic receptors in DM patients to restore or relieve SG-related complications, such as SG infection control and saliva flow promotion^13^. This study will provide primary knowledge of the two main purinergic receptors, P2X7R and P2X4R, which are commonly found in the SG, to pave the way for future research on the development of alternative methods of oral disease management. In this study, we aimed to mimic the T2DM condition in a mouse model by providing mice with a high-fat diet (HFD) and administering streptozotocin (STZ) to evaluate the morphological alterations and expression of P2X7R and P2X4R in the submandibular glands (SMGs).

## Results

### Effect of DM on mice body weight and per day diet, energy, and fat intake

**Figure 1:**
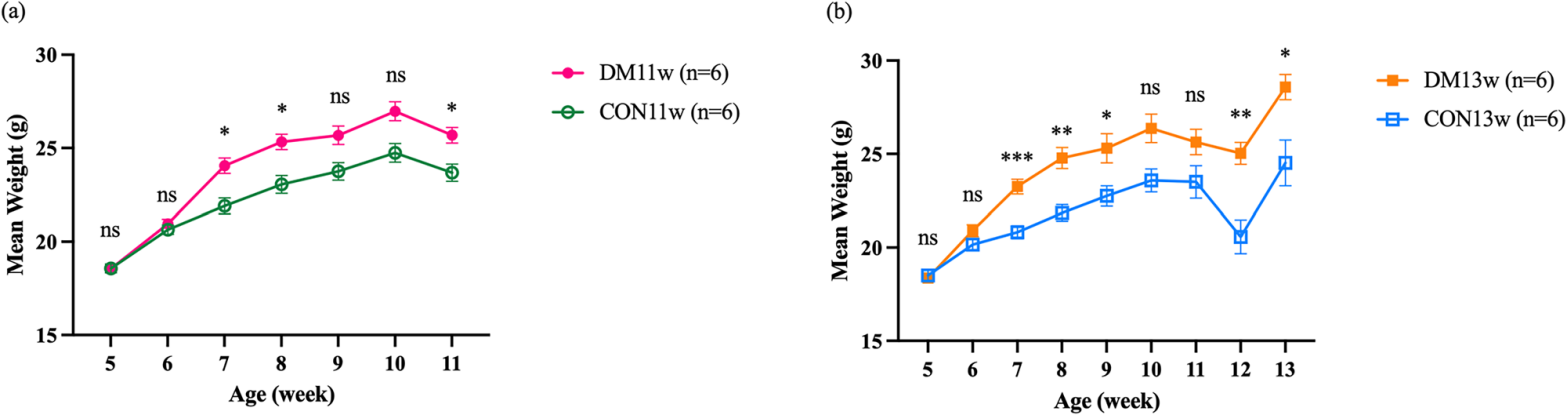
Mean body weights of the 24 mice in the CON and DM groups: (**a**) Weekly mean body weight of the CON11w (n = 6) and DM11w (n = 6) groups throughout the experiment. (**b**) Weekly mean body weight of the CON13w (n = 6) and DM13w (n = 6) groups throughout the experiment. The data were presented as the means ± standard error of the mean (SEM) (*p < 0.05, **p < 0.01, ***p < 0.001 and *ns* not significant).

**Figure 2:**
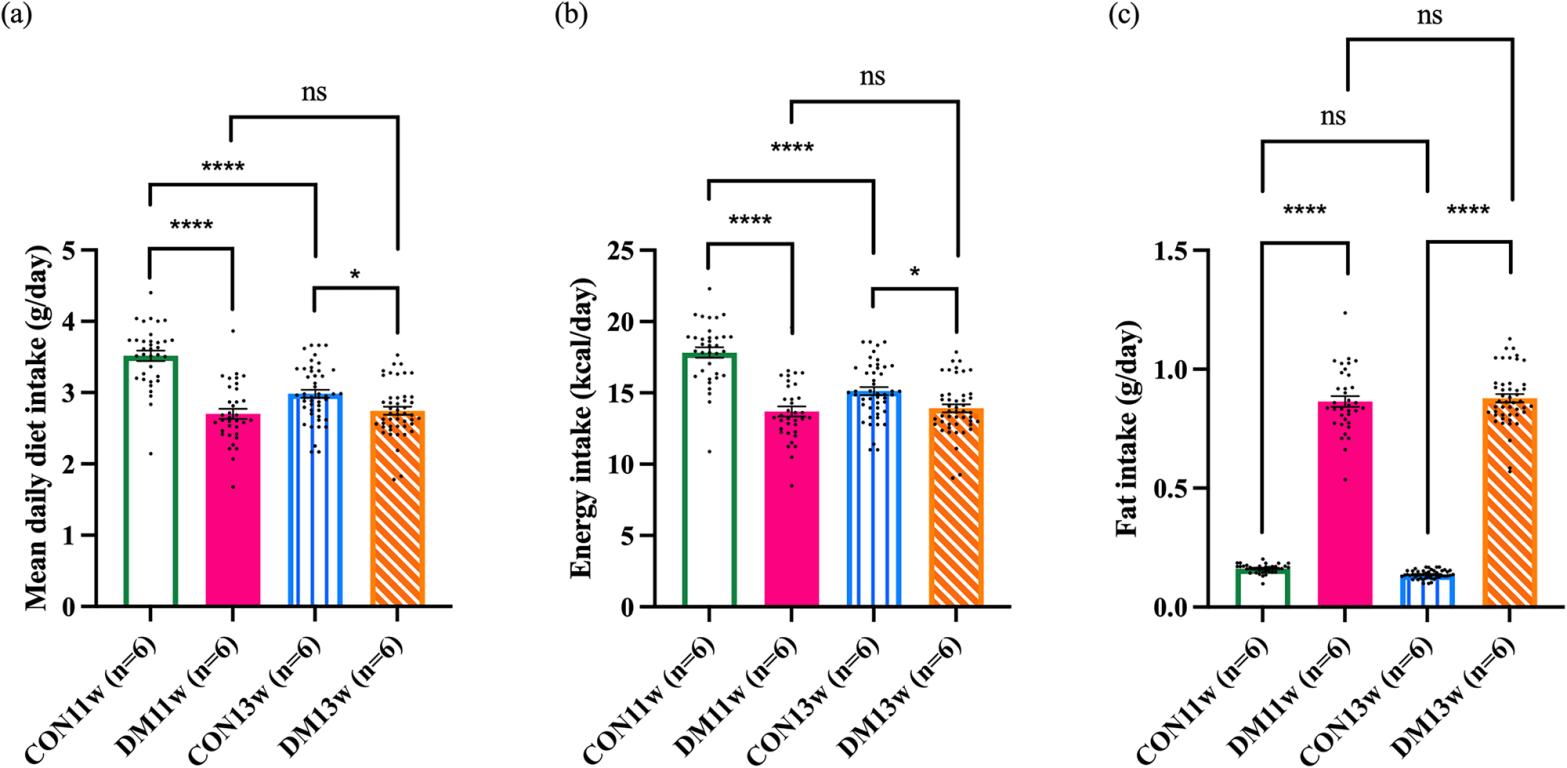
(**a**) Mean daily diet consumption of the 24 mice in the CON and DM groups throughout the experiment. (**b**) Daily mean energy intake of the CON and DM groups throughout the experiment. (**c**) Daily mean fat intake of the CON and DM groups throughout the experiment. Dots in the graphs represent daily values for 35 days of the CON11w and DM11w groups, and for 47 days in the CON13w and DM13w groups. The data were presented as the means ± SEM (*p < 0.05, ****p < 0.0001 and *ns* not significant).

At the beginning of the experiment, there was no significant difference in body weight among the groups. Throughout the experiment, the body weights of all groups tended to increase compared to their body weights in the first week of the experiment. Towards the end of the experiment, the body weights of the mice in the DM groups (i.e., DM11w and DM13w) were significantly higher than those in the CON group (i.e., CON11w and CON13w) (*p* < 0.05) (Fig. 1a,b). The DM group had a significantly lower daily consumption and energy intake than the CON group (*p* < 0.001 and *p* < 0.01, respectively) (Fig. 2a,b). However, fat consumption in the DM group was significantly higher than that in the CON group (*p* < 0.001) (Fig. 2c).

### Effect of DM on blood glucose level

**Figure 3:**
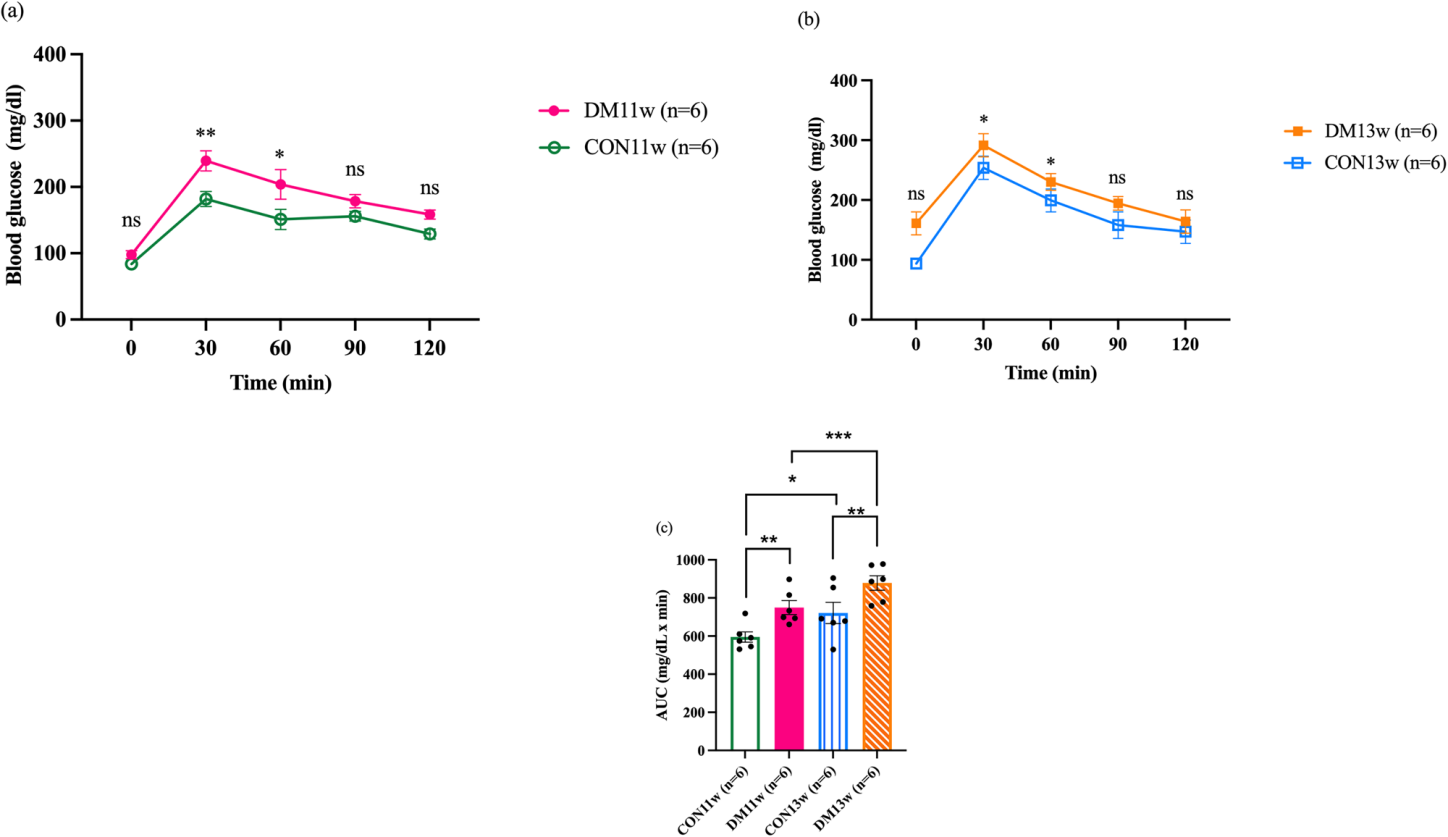
The OGTT and area under the curve (AUC) of the 24 mice in the CON and DM groups before sacrifice: (**a**) The 2nd OGTT compared between the CON11w (n = 6) and DM11w (n = 6) groups. (**b)** The 4th OGTT compared between the CON13w (n = 6) and DM13w (n = 6) groups (**c**) The 2nd AUC of the CON11w (n = 6) and DM11w (n = 6) groups and the 4th AUC of the CON13w (n = 6) and DM13w (n = 6) groups. The data were presented as the means ± SEM (*p < 0.05, **p < 0.01, ***p < 0.001 and *ns* not significant).

An oral glucose tolerance test (OGTT) was conducted, and the area under the curve (AUC) was calculated in all groups to verify blood glucose levels and assess insulin sensitivity before euthanasia. The 2nd OGTT comparing the CON11w and DM11w groups and the 4th OGTT comparing the CON13w and DM13w groups indicated a significant increase in blood glucose levels, reaching its peak at 30 min and gradually declining until 120 min after glucose challenge (Fig. 3a,b). In the DM11w and DM13w groups, there was a significant increase in blood glucose levels at 30 min (*p* < 0.01) and 60 min (*p* < 0.05) compared to the CON11w and CON13w groups at the same time points (Fig. 3a,b). The AUC of the DM group was significantly higher than that of the CON group (*p* < 0.01). Moreover, CON13w and DM13w had significantly higher AUCs than CON11w (*p* < 0.05) and DM11w (*p* < 0.001) (Fig. 3c).

### Morphological modifications of mice pancreas

**Figure 4:**
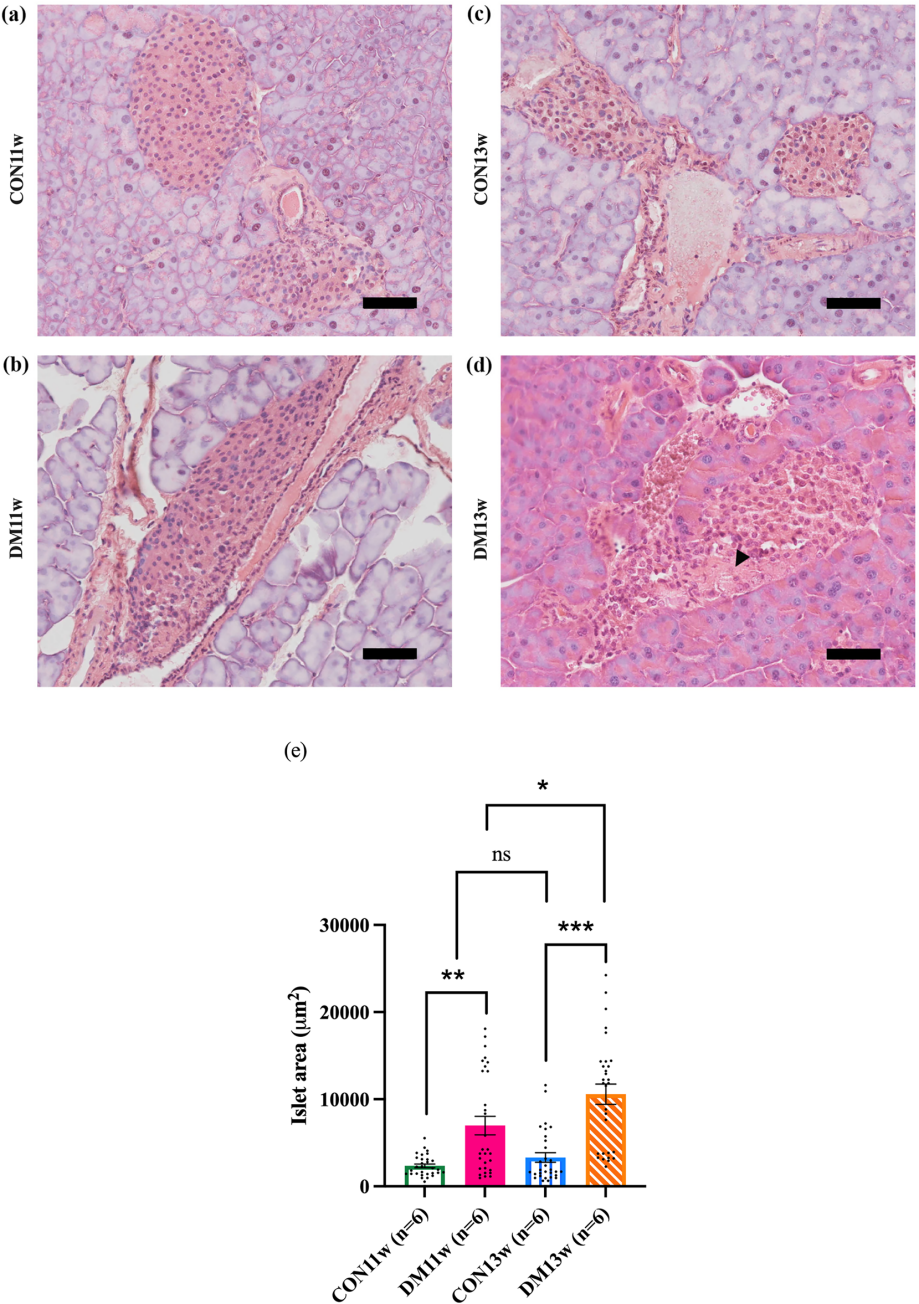
Hematoxylin–eosin (HE) staining of mice pancreas from the CON and DM groups. Magnification 200×: (**a**,**b**) The islet of Langerhans of CON11w (n = 6) and DM11w (n = 6) mice. (**c**,**d**) The islet of Langerhans of CON13w (n = 6) and DM13w (n = 6) mice. Amyloid accumulation was observed in DM13w mice (arrowhead). (**e**) Mean Islet area of the randomly five images of each mouse in CON group compared to that of the DM group. Dots in the graph represent 30 images in each group. The data were presented as the means ± SEM (*p < 0.05, **p < 0.01, ***p < 0.001 and *ns* not significant). Scale bars = 50 μm.

After the induction of diabetes, the pancreas in the DM group was damaged. Specifically, in the DM11w group, there was a disordered and loosely arranged structure of pancreatic parenchymal cells. In addition, mean size of the islets of Langerhans in the DM11w group were significantly larger than those in the CON11w group (Fig. 4a,b,e, *p* < 0.05). Conversely, the area of the pancreatic islets in the DM13w group was not markedly different from that in the CON group (Fig. 4c–e), as the area was replaced by amyloid accumulation (indicated by arrowhead). Furthermore, irregularly shaped and indistinct borders of the islets of Langerhans were observed in the DM13w group (Fig. 4d).

### Morphological change evaluation and morphometric analyses of SMG

**Figure 5:**
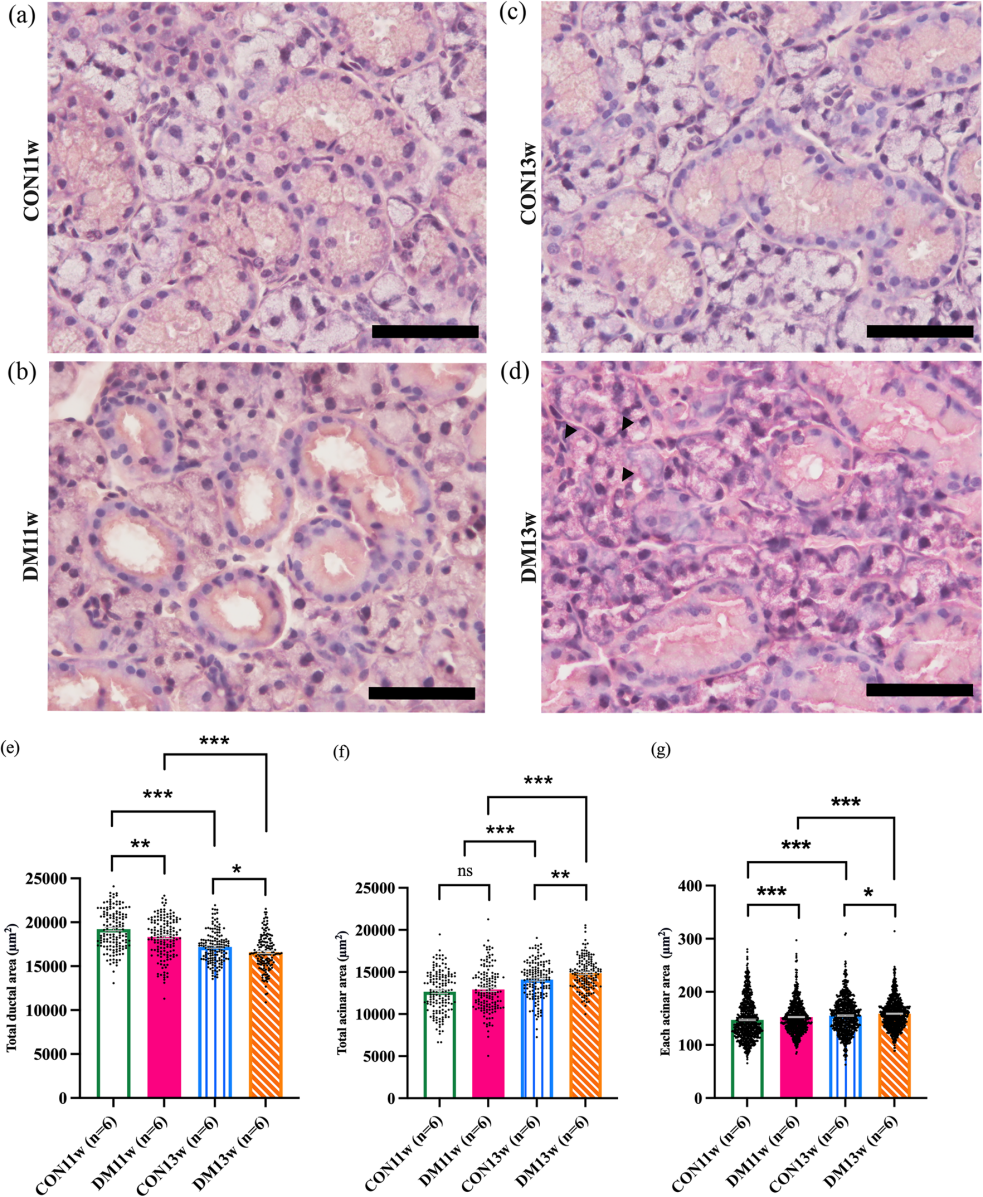
HE staining and morphometric analysis of the submandibular gland (SMGs) of the 24 mice from the CON and DM groups (Magnification; (**a**–**d**): 400×): (**a**–**d**) The histomorphology of the SMGs from the CON11w, DM11w, CON13w, and DM13w groups (n = 6 each), respectively. The arrowheads show the vacuolization of acinar cells. (**e**) The total ductal area of the CON group compared to that of the DM group measured from five SMG tissue sections per mouse, with ten randomly selected image fields per section of each group. Dots in the graph represent 300 images in each group. (**f**) The total acinar area of the CON group compared to that of the DM group measured from five SMG tissue sections per mouse, with ten randomly selected image fields per section of each group Dots in the graph represent 300 images in each group. (**g**) The area of acinar cells from the CON group compared to that of the DM group measured from randomly selected five acini in each image field of each mouse. Dots in the graph represent 1500 acini in each group. The data were presented as the means ± SEM (*p < 0.05, **p < 0.01, ***p < 0.001 and *ns* not significant). Scale bars = 50 μm.

The histomorphology of the SMG from the CON11w and CON13w groups exhibited a structured and dense arrangement with well-defined boundaries of acinar and ductal cells. Conversely, the DM11w group exhibited a disordered and loose arrangement of parenchymal cells, with a markedly enlarged ductal cell lumen. The acinar cells appeared irregularly shaped, dispersed, and densely packed (Fig. 5a,c). Additionally, the vacuolization of acinar cells (arrowheads), ductal cell enlargement (Fig. 5d), and infiltration of inflammatory cells (Supplementary Fig. 1d) were observed in the DM13w group with grade 3 according to Chisholm and Mason’s classification^18^. Conversely, the CON11w, DM11w and CON13w groups (Supplementary Fig. 1a–c) displayed mild lymphocyte infiltration, categorized as grade 1.

In the SMG, the total ductal area of the DM11w group was significantly lower than that in the CON11w group (*p* < 0.01). Similarly, the total ductal area in the DM13w group was significantly lower than that in the CON13w group (*p* < 0.05) (Fig. 5e). However, the total acinar area of the DM13w group increased significantly compared to that of the CON13w group (*p* < 0.01), while no significant difference was observed between the DM11w and CON11w groups (Fig. 5f). Similarly, the area of each acinar cell in the DM13w and DM11w groups was significantly higher than that in the CON13w (*p* < 0.05) and CON11w (*p* < 0.001) groups (Fig. 5g). Furthermore, the longer duration of diabetes induction in the DM13w groups resulted in a significantly lower total ductal area than the shorter duration of diabetes induction in DM11w groups (*p* < 0.001). Notably, the longer duration of diabetes induction in the DM13w groups led to a significantly lower total ductal area but a significantly higher total acinar and acinar cell area than those in the CON11w and DM11w groups (*p* < 0.001) (Fig. 5e–g).

### P2X4R and P2X7R localization and expression in the SMG

**Figure 6:**
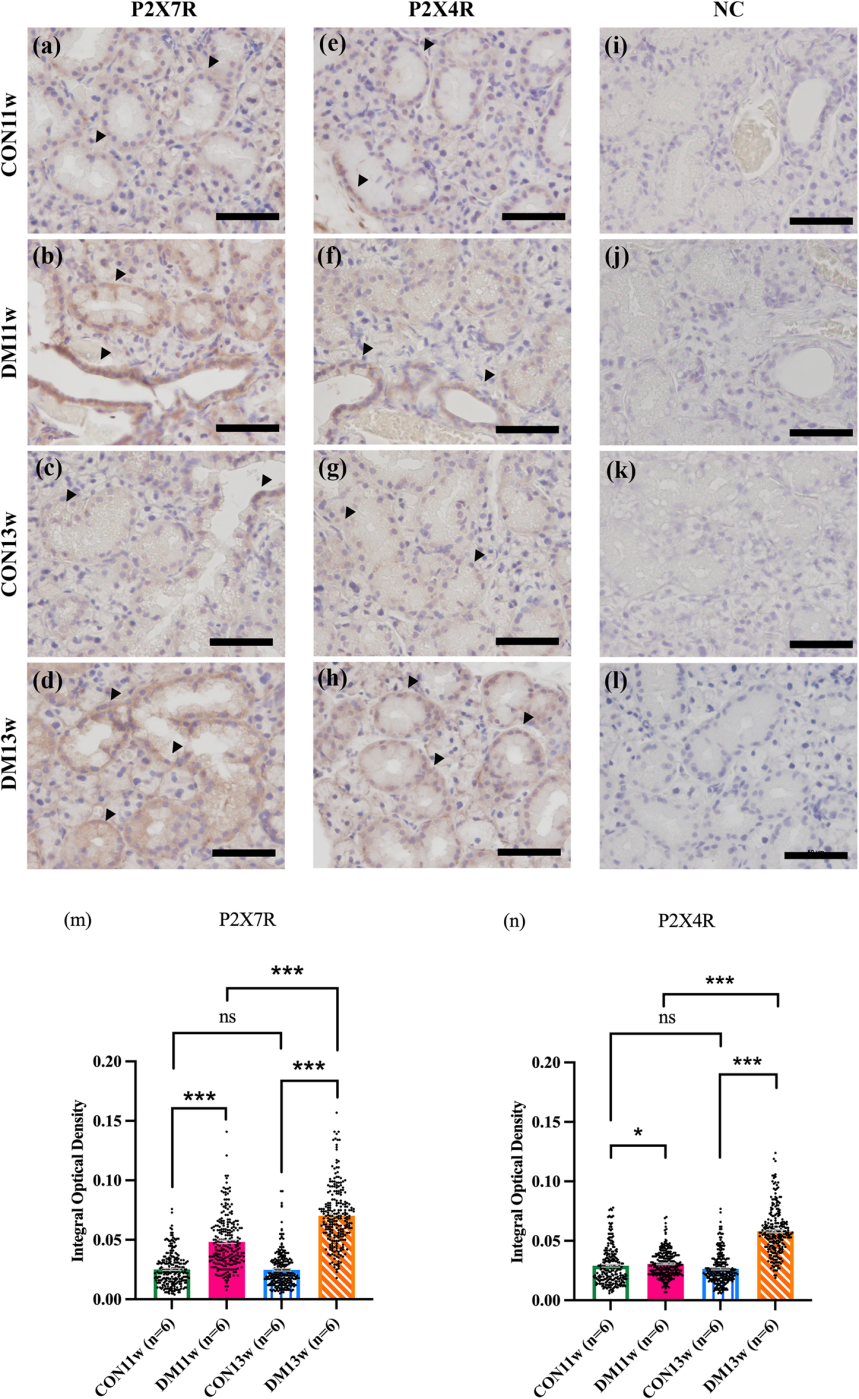
Immunohistochemical (IHC) staining and IHC-quantitative analysis of P2X7R and P2X4R in the SMGs of CON and DM groups: (**a**–**d**) IHC images of P2X7R expression in the CON11w, DM11w, CON13w, and DM13w groups. (**e**–**h**) IHC images of P2X4R in the CON11w, DM11w, CON13w, and DM13w groups. (**i**–**l**) IHC images of negative control (NC) in the CON11w, DM11w, CON13w, and DM13w groups. Scale bars = 50 μm. (**m**) The P2X7R area in the CON11w, DM11w, CON13w, and DM13w groups measured from 20 random fields of 300 × 300 pixels (0.05 × 0.05 mm) of two sections from each mouse. Dots in the graph represent 240 images in each group. (**n**) The P2X4R area in the CON11w, DM11w, CON13w, and DM13w groups measured from 20 random fields of 300 × 300 pixels (0.05 × 0.05 mm) of two sections from each mouse. Dots in the graph represent 240 images in each group. The data were presented as the means ± SEM (*p < 0.05, ***p < 0.001 and *ns* not significant).

Immunohistochemical analyses of the SMG revealed active staining of P2X4R and P2X7R localized on the basolateral part of acinar cells, with a higher intensity in the granular duct, which is the major duct found in the mouse SMG, exhibiting a similar receptor distribution pattern. Interestingly, the basal area of the striated ducts exhibited a stronger staining intensity than that of the other ductal types (Fig. 6b,f; arrowheads). CON11w and CON13w exhibited lower intensities of P2X7R and P2X4R active staining in SMG parenchymal cells. However, P2X7R and P2X4R expression in the acinar and ductal areas from the SMG of DM13w tended to increase compared to that in the CON groups, and their expression was significantly higher than that in DM11w for both receptors (Fig. 6a–h). Semi-quantitative IHC images revealed a significant increase in P2X7R and P2X4R in the DM13w group compared to that in the DM11w (*p* < 0.001) and CON13w (*p* < 0.001) group. Similarly, in the DM11w group, there was a significant increase in P2X7R and P2X4R compared to that in the CON11w group. No significant difference was observed between the CON11w and CON13w groups in terms of P2X7R and P2X4R expression (Fig. 6m,n).

### P2X7R and P2X4R expression in the SMG

**Figure 7:**
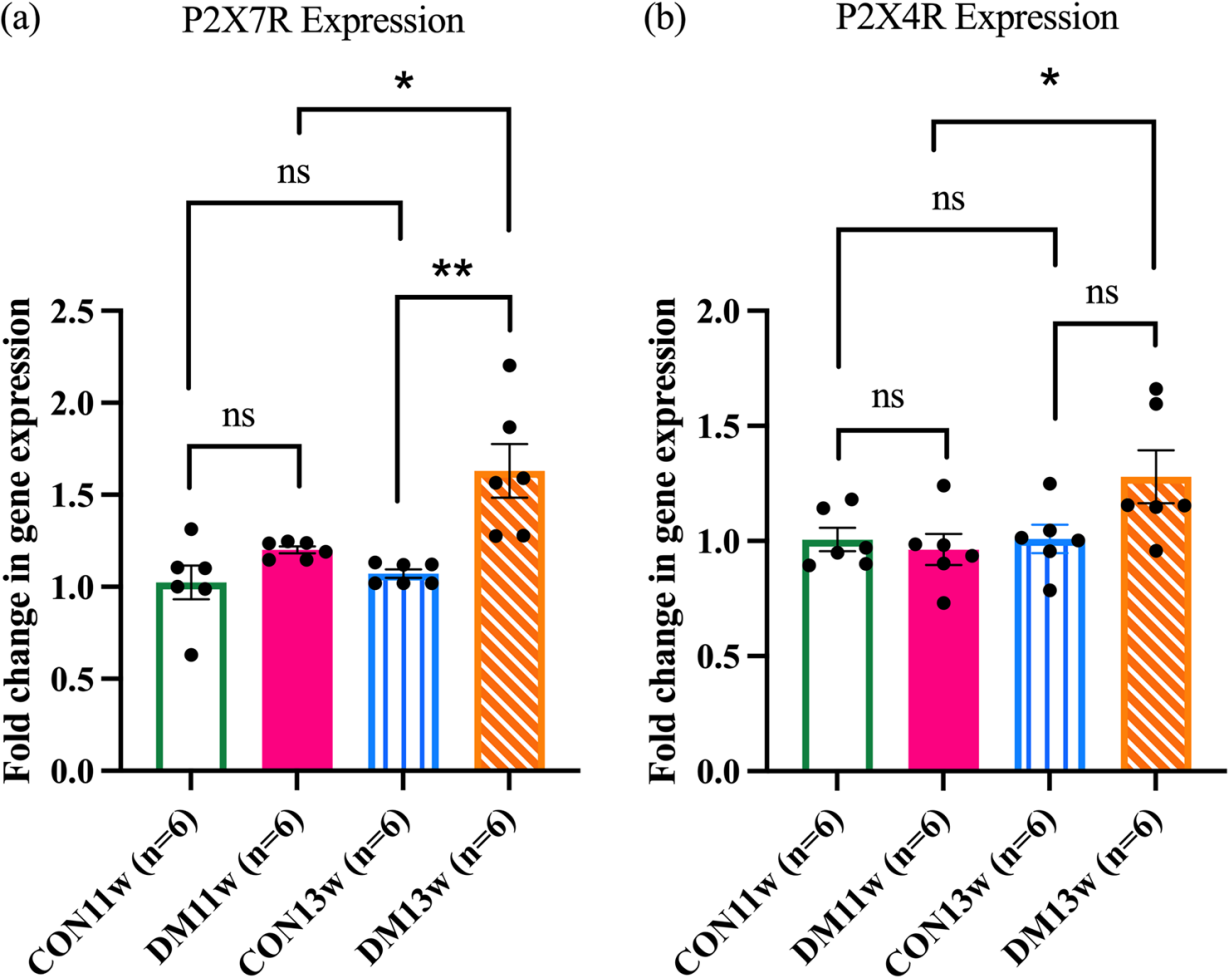
RT-qPCR analysis of P2X7R and P2X4R in the SMG of the CON and DM groups; (**a**) P2X7R mRNA expression in the CON11w, DM11w, CON13w, and DM13w groups (n = 6 each). (**b**) P2X4R mRNA expression in the CON11w, DM11w, CON13w, and DM13w groups (n = 6 each). The data were presented as the SEM (*p < 0.05, **p < 0.01 and *ns* not significant).

RT-qPCR analysis was performed to confirm the immunohistochemistry results and to evaluate the relative mRNA expression levels of P2X7R and P2X4R. The results revealed significantly higher gene expression of P2X7R in the DM13w group compared to the CON13w (*p* < 0.01) and DM11w groups (*p* < 0.05). However, there was no significant difference in P2X7R expression between the CON11w, DM11w, and CON13w groups (Fig. 7a). The increased P2X4R gene expression was found in DM13w compared with DM11w. Similar to P2X7R, P2X4R expression did not differ significantly between the CON11w and DM11w groups (Fig. 7b).

## Discussion

T2DMis a serious global health concern^19^ that is characterized by a progressive decline in insulin secretion, and increased peripheral resistance to insulin in various tissues, ultimately leading to hyperglycemia, which is associated with inflammation and metabolic stress^20^. This causes various diabetic complications, including oral diseases and salivary gland-related diseases^21^. Previous studies have focused on symptom-relieving methods or different types of receptors located on the SG^12^ to manage DM-related complications. However, this study is the first to observe the expression of P2X7R and P2X4R and their modifications in the SG after T2DM induction using a widely used HFD/STZ model^22^. As this model can mimic the pathological features of T2DM closely and can adjust the duration to modulate the severity and progression of diabetes which can investigate various aspects of diabetes development. However, inducing diabetes through a high-fat diet typically requires a prolonged feeding period before abnormalities develop. This may prolong timelines and increase the cost of materials^23^.

We also examined two different durations of T2DM induction to compare differences between the two groups by focusing on male mice to avoid hormonal fluctuations associated with estrous cycle in female mice^24^.

The DM group exhibited significantly higher body weights than the CON group at 5- to 9-weeks-old after being fed a HFD. Body weight decreased temporarily after STZ administration during the first 2 weeks (Fig. 1a,b). This can be attributed to the HFD being administered to the DM groups (HFD-32, CLEA, Tokyo, Japan) which is rich in highly saturated fat that affects body weight, and even insulin sensitivity^25,26^. Additionally, C57BL/6J mice are more prone to obesity than others^27^ due to the high susceptibility of central resistance to insulin activity and diminished anorexigenic peptide production from the hypothalamus^26^. After receiving STZ, leptin and adiponectin levels increase, affecting lipolysis and adipogenesis in mice, which corresponds to the results of this study^28^. The DM group consumed significantly less food than the CON groups (Fig. 2a). This can be explained by the feedback mechanisms of lipid messengers after continuous exposure to a diet containing a high fat percentage^29^. Although the dietary intake in the DM groups significantly decreased, the daily energy intake and overall weight gain increased significantly, which correlated with the higher total energy of the diet fed to the DM mice. After the induction of diabetes using HFD and STZ administration, hyperlipidemia develops, which is also related to the pathogenesis of T2DM^30,31^. The Area under the curve (AUC) from the OGTT graph generated before the mice were euthanized (2nd and 4th OGTT) demonstrated that the AUC of the DM group was significantly higher than that of the CON groups (Fig. 3a–c). This suggests impaired glucose tolerance, insulin sensitivity in the peripheral tissues, and impaired β cell function in the DM group. These results suggest that a HFD can induce oxidative stress that damages β cells in the islets of Langerhans in the pancreas, causing inflammation and eventually worsening fat and glucose metabolism^32,33^. To verify the T2DM model, the pancreas was observed and compared between the DM and CON groups. No major changes were observed in the pancreatic tissue at the onset of DM, except for enlarged islets of Langerhans area, which correspond to the increasing function of the pancreas at an early stage as detected in DM11w group. Prolonged DM resulted in marked histological alterations, including distorted and smaller islets of Langerhans, amyloidogenesis, vacuolization, and basophilic nuclei, which are signals of the cell apoptotic process^34–36^ which can be observed in DM13w group (Fig. 4a–d).

Alterations in SG function and histomorphology caused by T2DM have been previously established. Prior research has revealed a correlation between unusual histopathological findings and SG malfunction in rodents^37^ as well as morphological modifications in the presence of T2DM. However, a definitive understanding of the morphological changes that occur in the SG remains controversial. Our findings demonstrated signs of atrophic changes that the mean each acinar area in the DM groups were significantly larger than that in the CON groups. Whereas, the total acinar area of DM13w was larger when compared to the CON13w groups, although there was no significant difference between the DM11w and CON11w group. This could be attributed to the elevation of fibrous stromal tissue between the parenchymal areas in the early stages of T2DM, which corresponded with the results observed in the DM11w group (Fig. 5b). During a longer period of diabetes induction, acinar cells can regenerate and enlarge to compensate for degeneration. We observed that the total ductal areas in the DM11w and DM13w groups were significantly smaller than those in the CON11w and CON13w groups; however, a dilated lumen was observed. Additionally, the ductal area in the DM13w group was smaller than that in the DM11w group. Vacuolization (Fig. 5d, arrowheads) and Grade 3 of inflammatory cell infiltration (Supplementary Fig. 1d) were observed in the DM13w, indicating the presence of low-grade inflammation in T2DM^38^ compared to the CON11w, DM11w and CON13w groups (Supplementary Fig. 1a–c) which were defined as Grade 1 classification. In long-term T2DM conditions, as observed in the DM13w mice in this study, the ductal area is replaced with fibrous or fatty tissue, leading to a failure to regenerate acinar cells in the SG^39^.

In addition, several studies have investigated purinergic receptors in various organs affected by T2DM, including the retina, nephrons, microglia, neurons, and gastrointestinal organs^40,41^. No evidence has been found regarding inflammatory purinergic receptors in the SG after the development of T2DM. In this study, we examined the P2X7 and P2X4 receptors, which are the predominant subtypes found in the SG. Immunohistochemical staining revealed a similar pattern for the expression of P2X7R and P2X4R (Fig. 6a–l). There is some evidence suggesting the coexpression of P2X7R and P2X4R, although the precise interaction between them remains unknown^40^. A positive interaction between these receptors has been reported^42^, which may explain the corresponding patterns of P2X7R and P2X7R distribution. Purinergic receptor expression was observed in both acinar and ductal cells, with a greater intensity of P2X7R and P2X4R gene expression observed in ductal cells. We observed reduced P2X7 receptor expression in the CON11w and CON13w groups. Generally, P2X7R participates in the initial stages of inflammation, and its expression increases in response to macrophages, further aggravating inflammation^43–45^. A previous study on the retina also reported that P2X7R and P2X4R are associated with the progression of diabetic retinopathy^43^ which is related to our results suggested that the expression of P2X7 receptors was distinctly observed in the DM11w group and became more intense in the DM13w group. The significantly higher expression of P2X7R and P2X4R in T2DM could be explained by the response of purinergic receptors to a stressful environment. When tissues are exposed to pathological conditions, they are stimulated to release ATP, which binds to purinergic receptors. Activation of purinergic signaling pathways stimulates the production of cytokines leading to ion transportation^46^, followed by activation of inflammatory pathways^43,47^. Although a previous study demonstrated that P2X4R overexpression was associated with IL-6 cytokine release and inflammation^48^, the precise role of P2X4R under stress conditions and in the inflammatory process remains unclear. Evidence suggests that P2X4R plays a role in the initiation of inflammation under extracellular ATP conditions^49^. Our results revealed that P2X4R expression was significantly elevated in the DM13w group compared to the DM11w group (Fig. 7b). Thus, we assumed that P2X4R in the SG might have similar roles in the other organ (e.g. retina)^43^.

RT-qPCR was used to confirm these hypotheses by analyzing the mRNA transcription levels of P2X7R and P2X4R, and the results correlated with the IHC images. The DM13w group exhibited a significantly higher P2X7R mRNA level compared to the CON13w and DM11w groups. Furthermore, the expression of P2X4R was significantly higher in the DM13w group when compared to that in the DM11w group. P2X4R expression in the DM groups demonstrated a progressive time-dependent trend, but there was no significant difference between the DM13w, DM11w, CON13w and CON11w groups (Fig. 7a,b). Although RT-qPCR is a useful method for gene detection, its main limitation is that it requires a precise known target gene sequence^50^. To date, the function and biological structure of P2X4R remain ambiguous, leading to unfavorable RT-qPCR results that do not support our hypothesis suggesting that P2X4R expression correlates with P2X7R expression in DM11w mice (Fig. 7b). Therefore, further investigation is required to gain a deeper understanding of P2X4R. However, our RT-qPCR results for P2X4R in DM13w and P2X7R in all groups were related to the IHC staining images.

Based on our results, the structural characteristics and location of both P2X7R and P2X4R in the SG exhibited a similar increasing trend in expression but remained relatively unaffected by T2DM. Nonetheless, our analysis of integral optical density (IntOD) from the IHC images and gene expression data obtained through RT-qPCR indicated a certain extent of alterations in response to T2DM. Additionally, recent studies have reported interactions between these two receptors, although the precise underlying mechanisms remain unclear^42,51^.

Along with the limitations mentioned above, we also did not investigate the function of the receptors and downstream molecular pathways of SMG in our T2DM model. Nevertheless, our study provides fundamental knowledge that can facilitate further research aimed at exploring novel alternative therapeutic methods for patients with SG impairment caused by diabetic complications.

## Conclusions

Our study demonstrated a significant association between T2DM and changes in SG, showing notable alterations in SG structure and increase in P2X7 and P2X4 receptor levels in diabetic conditions. These finding not only highlight the complex relationship between diabetes and SG but also suggest possibilities for treatment development. By discovering the molecular mechanisms through which diabetes impacts the SG, our research provides valuable insights for potential therapeutic approaches. Targeting purinergic signaling pathways based on our findings could pave the ways for the development of novel treatments to alleviate diabetic complications.

## Materials and methods

### Animals and experimental design

Twenty-four 5-week-old male C57BL/6J mice (n = 24) were obtained from Sankyo Labo Service (Tokyo, Japan). The animal experiments conducted in this study were approved by the Institutional Animal Care and Welfare Committee (A2022-090A) of Tokyo Medical and Dental University (TMDU), followed the Animal Care Standards of TMDU in the management and handling of experimental animals, and complied with the ARRIVE guidelines.

Three mice were housed per cage under controlled temperature, humidity, and lighting (12-h light–dark cycle) conditions, and provided with ad libitum access to water throughout the experiment. After a 5-day acclimation period, the mice were randomly assigned to two groups: CON and DM. The control (CON) group was fed a standard rodent diet (SD) (CE-2, CLEA, Tokyo, Japan), which provided 3.402 kcal/g total energy and 4.6% fat kcal. In contrast, the DM group received a high-fat diet (HFD) (HFD-32, CLEA, Tokyo, Japan), which contained 5.076 kcal/g total energy and 32% fat kcal (details of the ingredients for SD^52^ and HFD^53^ are shown in Supplementary Tables 1a and b, respectively). The groups were further categorized as CON11w and DM11w, designated for mice that were 11-weeks-old or observed for 6 weeks, respectively, or as CON13w and DM13w, designated for mice that were 13-weeks-old or observed for 8 weeks before euthanasia.

**Figure 8:**
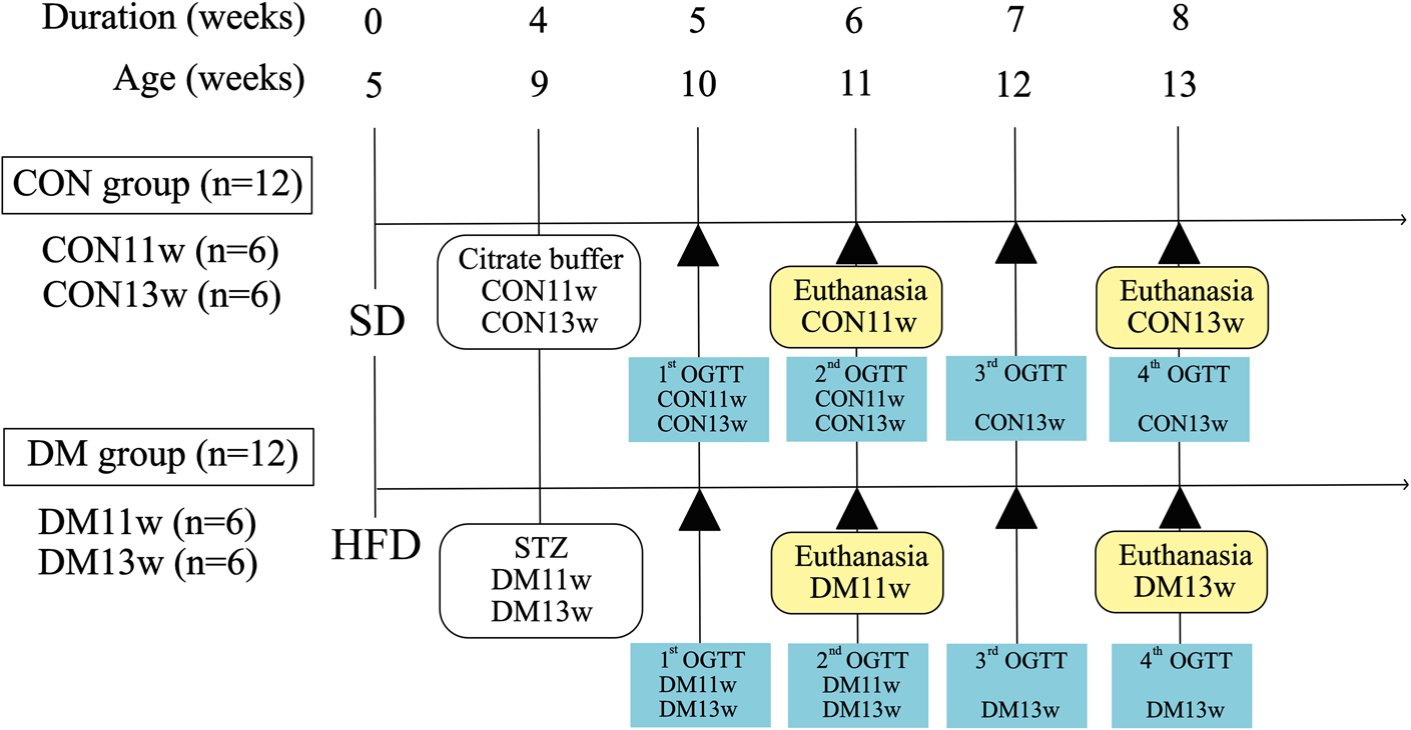
Experimental design. Twenty-four 5-week-old male C57BL/6J mice (n = 24) were randomly divided into four groups: two control groups (CON11w and CON13w, n = 6 each) and two type 2 diabetes mellitus groups (DM11w and DM13w, n = 6 each). The CON group was fed a standard diet (SD) and the DM group was fed a high-fat diet (HFD). During the 4th week of dietary establishment, the DM11w (n = 6) and DM13w (n = 6) mice were treated with low doses of streptozotocin (STZ) solution. The CON11w (n = 6) and CON13w (n = 6) mice were given a vehicle (citrate buffer) only. After STZ injection, an oral glucose tolerance test (OGTT) was performed weekly. Finally, at the 6th and 8th week of the experiment, all mice were euthanized.

In the 4th week of dietary establishment, DM11w and DM13w mice were injected intraperitoneally with a low dose (40 mg/kg) of streptozotocin (STZ) (Sigma-Aldrich, St. Louis, MO, USA) diluted in 0.05 M citrate buffer solution (pH 4.5)^54^, while CON11w and CON13w mice were intraperitoneally administered a vehicle (citrate buffer). All mice were fasted for 4 h before injection in accordance with the National Institutes of Health (NIH) Diabetic Complications Consortium protocol^55^. An oral glucose tolerance test (OGTT) was performed weekly after STZ administration. Diet consumption was monitored daily, excluding the acclimation and fasting periods before OGTT measurement, for 35 days in CON11w and DM11w groups, whereas the CON13w and DM13w groups were observed for 47 days. diet consumption and twice-weekly body weight monitoring were performed throughout the experiment. At 6 and 8 weeks, all mice were euthanized under inhalation anesthesia (isoflurane; Wako, Japan), and their SMGs were immediately dissected for further analysis using real-time quantitative reverse transcription polymerase chain reaction (RT*-*qPCR), immunohistochemistry (IHC), and hematoxylin–eosin (HE) staining. The pancreas was collected from each mouse to confirm histological alterations after diabetes induction via HE staining. A summary of the experimental design is provided in Fig. 8.

### The oral glucose tolerance test (OGTT)

The OGTT was conducted weekly from the time of STZ administration until the mice were euthanized to confirm complete induction of T2DM. All of the mice were fasted overnight for 18 h prior to the test^56^. Baseline blood glucose levels were measured at 0 min, and a 10% glucose solution prepared with d-(+)-glucose (Nacalai Tesque, Japan) in distilled water was orally administered at a dose of 1 g/kg body weight^57^ and a volume of 10 μl/g body weight^58^. Blood glucose levels were recorded at 0, 30, 60, 90 and 120 min^59^ using the ACCU-CHEK Guide Blood Sugar Test Kit (Roche Diagnostics, Indianapolis, IN, USA). Blood samples (5 μl) were obtained by tail pricking. The area under the curve for blood glucose (AUC_glu_) were calculated using GraphPad Prism 9 software (GraphPad Software Inc., La Jolla, CA, USA).

### Histological preparation of SMG and pancreas

The right SMGs and pancreas of sacrificed mice were immediately fixed with 4% paraformaldehyde (Mildform^®^; Wako Pure Chemical Industries, Osaka, Japan) for 24 h. To obtain histological images, the samples were dehydrated with graded ethanol and embedded in paraffin wax blocks using an embedding machine (RH-12DM; Sakura Finetek Japan, Japan) following the standard protocol. The embedded tissues were then serially sectioned into 5-μm-thick pieces using a microtome sectioning machine (Leica Biosystems, Nussloch, Germany) for mounting on glass slides. Finally, right SMGs and pancreatic tissues were subjected to HE staining and the IHC process.

### Histomorphological evaluation

To stain the sections of the SMG and pancreas with HE, the sections were first deparaffinized with xylene, rehydrated by immersion in sequentially diluted alcohols for 3 min each, and rinsed with water for 3 min. The tissues were then stained with hematoxylin for 12 min, followed by eosin for 1 min 15 s. The histological characteristics of the SMG and pancreas were observed under a light microscope (Eclipse 80i; Nikon, Tokyo, Japan) at magnifications of 400× and 200×, respectively. The images were captured using a digital camera (DS-Ri1; Nikon, Kanagawa, Japan).

### Morphometric analyses of the SMG

Five SMG tissue sections per mouse, with ten randomly selected image fields per section, were captured at 400× magnification for morphometric analysis. The total area of ductal cells, the total area of acinar cells, and the size of each acinar cell were analyzed using Fiji ImageJ2 software (version 2.9.0; National Institutes of Health, Bethesda, MD, USA). Prior to measuring the total ductal and acinar areas, the stromal and parenchymal areas were identified. Specifically, the stromal area was excluded from the field by adjusting the brightness using a color threshold function. The total ductal area was determined by summing the ductal areas manually using a freehand selection tool. The total acinar area was indirectly calculated by subtracting the total ductal area from the parenchymal area, as described by the following equation:

$${\text{Total parenchymal area}}\;(\upmu {{\text{m}}^2}) = {\text{ image field area}}\;(\upmu {{\text{m}}^2}) \, - {\text{total stromal area}}\;(\upmu {{\text{m}}^2}) = {\text{ total ductal area}}\;(\upmu {{\text{m}}^2}) + {\text{ total acinar area}}\;(\upmu {{\text{m}}^2})$$

Five acini were randomly selected and measured in each image field. To determine the area of a single acinar cell, the area of the acinus was divided by the number of nuclei within that acinus, following the protocol described in a previous study^60^.

Furthermore, the inflammatory status in salivary gland tissues from each group was evaluated by the degree of lymphocyte infiltration using the Chisholm and Mason classification system^18^. This system assigns grades based on the severity of infiltration: Grade 0: No lymphocyte infiltration, Grade 1: slightly presence, Grade 2: moderately presence, Grade 3: one focus of at least 50 aggregated lymphocytes and Grade 4: multiple foci of lymphocyte aggregation.

### Confirmation of histological change in pancreas after diabetes induction

The cellular characteristics of the parenchyma cells and islets of Langerhans were observed to validate the T2DM model. Five randomly chosen images from each mouse were examined at 200× magnification. Islet areas within each image were quantified using the freehand selection tool in Fiji ImageJ2 software (version 2.9.0; National Institutes of Health, Bethesda, MD, USA). The mean islet area was subsequently calculated as μm^2^. The islet quantification approach was modified from a previous investigation^61^.

### Immunohistochemical (IHC) staining

The expression and localization of P2X7R and P2X4R in the SMG were analyzed by immunohistochemistry using an avidin/biotin-based peroxidase system. Formalin-fixed paraffin-embedded tissues were deparaffinized and rehydrated using xylene and a graded ethanol series, respectively. Antigen retrieval was performed overnight in a water bath set at 60 °C with sodium citrate buffer solution (pH 6.0). The tissue sections were then washed twice with 0.1 M phosphate-buffered saline (PBS) pH 7.4 for 5 min, followed by permeabilization in PBS containing 0.2% Triton-X100 for 10 min at room temperature (RT). Hydrogen peroxide (H_2_O_2_) in methanol was used as a quenching solution for 30 min at RT. Next, the sections were rinsed twice with distilled water for 5 min and then blocked for non-specific binding in normal goat serum solution for 30 min at RT. The sections were then incubated with primary antibodies against P2X7R (#APR-004; Alomone Laboratories, Jerusalem, Israel) and P2X4R (#APR-002; Alomone Laboratories, Jerusalem, Israel) in PBS (diluted for P2X4R = 1:200, P2X7R = 1:500) overnight at 4 °C. After applying the primary antibodies and rinsing thrice with PBS for 5 min, the following procedures were performed using reagents from VECTASTAIN Elite ABC-HRP Kit (PK-6100, Vector Laboratories, USA). The sections were incubated with secondary antibodies against rabbit IgG, which was provided in the kit, for 30 min at RT and rinsed thrice with PBS for 5 min. Finally, the VECTASTAIN Elite ABC reagent was applied for 30 min at RT, and the sections were washed thrice with PBS for 5 min before proceeding to the next steps.

Next, tissues were treated with 3,3-diaminobenzidine (DAB substrate kit; #ab64238; Abcam, Cambridge, MA, United States) for 40 s. After rinsing the sections with distilled water, Mayer’s hematoxylin (Fujifilm Wako Pure Chemical Corp., Osaka, Japan) was used for nuclear staining, followed by rinsing with tap water for 15 min. Before mounting, sections were dehydrated using a graded ethanol series and xylene. Finally, the sections were mounted using Mount-Quick (Hokkaido Sangyo, Japan) and visualized under a light microscope (Eclipse 80i; Nikon, Tokyo, Japan) equipped with a digital camera (DS-Ri1; Nikon, Kanagawa, Japan) at 400× magnification. Two sections were observed per mouse, and 20 random fields of 300 × 300 pixels (0.05 × 0.05 mm) were captured^60^. To perform a semi-quantitative analysis of P2X7R and P2X4R expression in the IHC images, the integral optical density (IntOD) of P2X4R and P2X7R was measured in SMG sections using Fiji ImageJ2 software (version 2.9.0; National Institutes of Health, Bethesda, MD, USA).

### Reverse transcription-quantitative polymerase chain reaction (RT-qPCR)

The remaining SMGs of each mouse were excised and immediately frozen at − 80 °C for reverse transcription quantitative polymerase chain reaction (RT-qPCR). The tissues were homogenized using a BioMasher II (Nippi Inc., Tokyo, Japan), and total mRNA was extracted using Sepasol RNA I Super G reagent (Nacalai Tesque, Japan), following the manufacturer’s protocol. Briefly, the samples were homogenized in 1 ml of Sepasol RNA I Super G and incubated at RT for 5 min. Thereafter, 200 μl of chloroform (Fujifilm Wako Pure Chemical Corp., Osaka, Japan) was added to the tubes, which were subsequently shaken and centrifuged at 12,000×*g* for 15 min at 4 °C. Next, the upper aqueous phase was transferred to new tubes and combined with 500 μl of 2-propranolol (Fujifilm Wako Pure Chemical Corp., Osaka, Japan). After resting at RT for 10 min and centrifuging at 12,000×*g* for 10 min at 4 °C, the supernatant was removed. To clear the RNA pellets, 1 ml of 75% ethanol (Fujifilm Wako Pure Chemical Corp., Osaka, Japan) was added to the tubes and centrifuged at 12,000×*g* for 5 min at 4 °C. Finally, the RNA concentration at 260 nm was determined using a spectrophotometer (Beckman DU^®^ 640; GMI, Minnesota, USA). Complementary DNA (cDNA) was synthetized from 1 μg of total RNA with reverse transcription random primers using ReverTra Ace™ qPCR RT Master Mix (TOYOBO, Osaka, Japan), following the manufacturer’s instructions. RT-qPCR was performed using an Applied Biosystems 7500 Real-Time PCR System (Thermo Fisher Scientific Inc., Waltham, MA, USA) with TB Green^®^ Premix Ex Taq™ II (Tli RNase H Plus) kits (TaKaRa Bio, Otsu, Japan). The PCR mixture per reaction included 25 ng of cDNA template, TB Green^®^ Premix Ex Taq™ II (2×), PCR forward and reverse primers (10 μm each), ROX reference dye II (50×), and sterile purified water (RNase-free water, non DEPC; BioDynamics, Tokyo, Japan). All data were normalized to GAPDH mRNA. Amplified oligonucleotide primers specific for mouse GAPDH, P2X7R, and P2X4R were purchased from Thermo Fisher Scientific Inc. (Waltham, MA, USA). The primer sequences used in this study are listed in Table 1.

**Table 1:** Primer sequences used in RT-qPCR process.

Gene	Forward primer sequence (5′–3′)	Reverse primer sequence (5′–3′)	GenBank No.*
GAPDH	GCATCTTCTTGTGCAGTGCC	TACGGCCAAATCCGTTCACA	AY618199.1
P2X7R	GCACGAATTATGGCACCGTC	CCCCACCCTCTGTGACATTC	AJ009823.1
P2X4R	CGGGCTTTCCTGTTCGAGTA	AAAGTTGGCGTTGGCGTAAG	JX674050.1

*GenBank accession numbers of sequences used for primer design.

Each amplification cycle consisted of an initial denaturation stage at 95 °C for 30 s, followed by 40 cycles of heating at 95 °C for 5 s, annealing at 60 °C for 34 s, 95 °C for 15 s, 60 °C for 60 s, and 95 °C for 15 s. Each assay was repeated three times. Relative mRNA expression levels were calculated using the comparative Ct method (2^−ΔΔCt^) with GAPDH serving as an internal control. To validate these results, melting curves were obtained, which displayed a single normal peak. The mRNA expression levels were reported as n-fold change relative to the mRNA expression in the control groups.

### Statistical analysis

Statistical analyses were performed using GraphPad Prism version 9 (GraphPad Software Inc., La Jolla, CA, USA). The data were presented as the means ± standard error of the mean (SEM). Normal distribution of the data was assessed using the Shapiro–Wilk test. To compare mice body weight, diet consumption, calorie intake, fat intake and AUC data between CON11w/DM11w and CON13w/DM13w, an unpaired two-tailed Student’s t-test was performed. To compare the total ductal area, total acinar area, and fold change of P2X4R and P2X7R expression among the CON11w, DM11w, CON13w, and DM13w groups, one-way analysis of variance (ANOVA), followed by Holm–Šídák’s multiple comparison test, was used. To compare the acinar area, mean islet areas and integral optical density of P2X4R and P2X7R expression data among the CON11w, DM11w, CON13w, and DM13w groups, the Kruskal–Wallis test, followed by Dunn’s multiple comparison test, was applied. Statistical significance was defined as *p* < 0.05.

### Supplementary Information

Supplementary Information.

## Data Availability

The datasets generated during and/or analyzed during the current study are available from the corresponding author on reasonable request.

## Abbreviations

DM: Diabetes mellitus
T2DM: Type 2 diabetes mellitus
P2X7R: P2X7 purinergic receptor
P2X4R: P2X4 purinergic receptor
SMG: Submandibular gland
