# Supplementary material for: P2X7R and P2X4R expression of mice submandibular gland in high-fat diet/streptozotocin-induced type 2 diabetes
Source: Sci Rep. 2024 May 13;14:10855. doi: 10.1038/s41598-024-60519-3 (PMC11091137; doi:10.1038/s41598-024-60519-3)
Supplement: Supplementary file 1 — Supplementary Information. [file 41598_2024_60519_MOESM1_ESM.pdf]

Supplementary Materials

P2X7R and P2X4R expression of mice submandibular gland in high-fat diet/streptozotocin-induced type 2 diabetes

Jiratchaya Srisutha[1,2], Ippei Watari[1], Masato Akakura[1], Minami Watanabe[1], Chidsanu Changsiripun[2] and Takashi Ono[1]
[1]Department of Orthodontic Science, Graduate school of Medical and Dental Sciences, Tokyo Medical and Dental University (TMDU), Tokyo 113-8510, Japan. [2]Department of Orthodontics, Faculty of Dentistry, Chulalongkorn University, Bangkok 10330, Thailand. Email: ippeiwatari@gmail.com

(a)

Ingredients	Percentage (%)
Milk casein	24.5
Saffllower oil (high oleic acid)	20
Powdered beef tallow	15.880
Maltodextrin	8.25
Lactose	6.928
Sucrose	6.750
Crystaline cellulose	5.5
Egg white	5
AIN93G mineral mix	5
AIN93 vitamin mix	1.4
L-cystine	0.43
Choline bitartrate	0.36
Tertiary butylhydroquinone	0.002
**Total**	**100.000**

(b)

Ingredients	Percentage (%)
Nitrogen-free extracts	47.20
Protein (whitefish, soybean and yeast)	26.46
Moisture	8.70
Ash	8.60
Fat (cereal germ, soybean oil)	5
Fiber (wheat bran, defatted rice bran, alfalfa meal)	4.04
**Total**	**100.000**

Supplementary materials. Table 1: Ingredients of diet used in this study. (a) Ingredients of high fat diet (HFD-32) (b) Ingredients of standard diet (CE-2). CLEA, Tokyo, Japan[1,2]

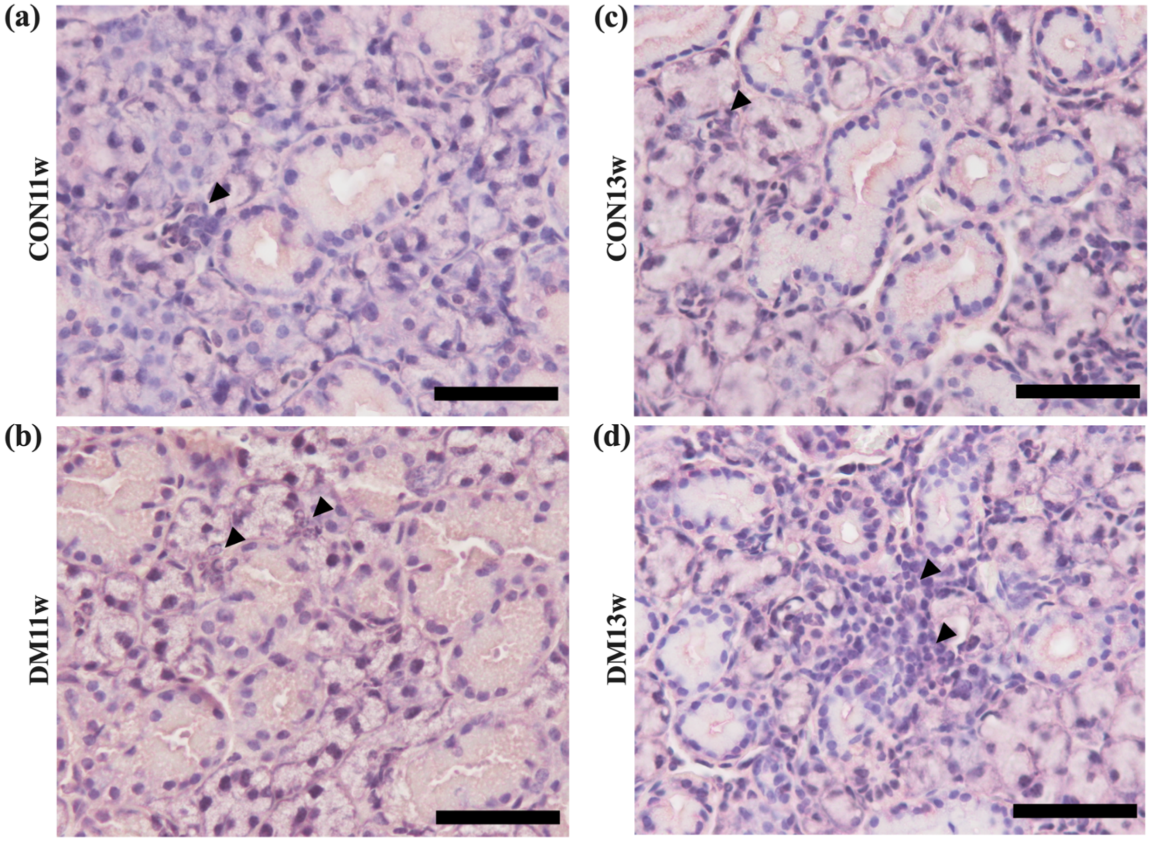

Supplementary materials. Figure 1: The inflammatory cells detected in SG parenchymal area of CON and DM groups (arrow heads) Magnification 400×. (a) CON11w group (b) DM11w group (c) CON13w (d) DM13w. Scale bars = 50 µm.

References

1 Ezaki, O. *For mice, rats, and hamsters CLEA Rodent Diet CE-2 (for rearing and breeding)*, <https://www.clea-japan.com/en/products/general_diet/item_d0030

2 Ezaki, O. *High Fat Diet 32*, <https://www.clea-japan.com/en/products/general_diet/item_d0080
